# Plasma peptidome profiling of acute hepatitis E patients by MALDI-TOF/TOF

**DOI:** 10.1186/1477-5956-9-5

**Published:** 2011-02-04

**Authors:** Shikha Taneja, Imran Ahmad, Somdutta Sen, Saravanan Kumar, Reena Arora, Vijay K Gupta, Rakesh Aggarwal, Krishnamoorthy Narayanasamy, Vanga S Reddy, Shahid Jameel

**Affiliations:** 1 Virology Group, International Centre for Genetic Engineering and Biotechnology, Aruna Asaf Ali Marg, New Delhi - 110067, India; 2 The Centre for Genomic Applications, Okhla Industrial Area (Phase III), New Delhi - 110020, India; 3 Plant Transformation Group, International Centre for Genetic Engineering and Biotechnology, Aruna Asaf Ali Marg, New Delhi - 110067, India; 4 Department of Gastroenterology, Army Hospital, Delhi Cantonment, New Delhi - 110010, India; 5 Department of Gastroenterology, Sanjay Gandhi Postgraduate Institute of Medical Sciences, Raebareli Road, Lucknow - 226014, India; 6 Sphaera Pharma Research and Development, 32, Sector 5, IMT Manesar, Haryana - 122051, India

## Abstract

**Background:**

Hepatitis E is endemic to resource-poor regions, where it manifests as sporadic cases and large waterborne outbreaks. The disease severity ranges from acute self-limited hepatitis with low mortality to fulminant hepatic failure with high mortality. It is believed that the host response plays an important role in determining the progression and outcome of this disease. We profiled the plasma peptidome from hepatitis E patients to discover suitable biomarkers and understand disease pathogenesis.

**Results:**

The peptidome (< 10 kDa) fraction of plasma was enriched and analyzed by mass spectrometry. A comparative analysis of the peptide pattern of hepatitis E patients versus healthy controls was performed using ClinPro Tools. We generated a peptide profile that could be used for selective identification of hepatitis E cases. We have identified five potential biomarker peaks with m/z values of 9288.6, 7763.6, 4961.5, 1060.572 and 2365.139 that can be used to reliably differentiate between hepatitis E patients and controls with areas under the receiver operating characteristic curve (AUROC) values of 1.00, 0.954, 0.989, 0.960 and 0.829 respectively. A number of proteins involved in innate immunity were identified to be differentially present in the plasma of patients compared to healthy controls.

**Conclusions:**

Besides the utility of this approach for biomarker discovery, identification of changes in endogenous peptides in hepatitis E patient plasma has increased our understanding of disease pathogenesis. We have identified peptides in plasma that can reliably distinguish hepatitis E patients from healthy controls. Results from this and an earlier proteomics study are discussed.

## Background

Hepatitis E, caused by infection with the enterically transmitted hepatitis E virus (HEV), is a common form of hepatitis in areas with poor sanitation and hygiene. It causes an acute self-limiting disease mainly in young adults with a low mortality of 0.2% to 1.0% in the general population. However, in some cases the disease progresses to fulminant hepatic failure (FHF) with high mortality. For reasons yet not determined, there is increased incidence and severity in pregnant women infected during the second and the third trimester with mortality rates between 15-25% [1,2]. Hepatitis E is endemic to large parts of Asia, Africa, the Mediterranean region, Mexico and South America, where it is responsible for outbreaks that are often large, and affect several hundred to several thousand people [3]. In disease-endemic areas, HEV infection also accounts for a large proportion of acute sporadic hepatitis in all age groups. It is estimated that about 2 billion people live in areas endemic for HEV [4].

The geographic prevalence of antibody to HEV is worldwide. In India, HEV infection is the most common cause of acute sporadic hepatitis and accounts for up to 50% of such cases among adults. In developed countries, the disease was initially found to occur almost exclusively among travellers to disease-endemic regions. Over the past few years, there has been an increase in the number of autochthonous cases in developed countries and evidence for a possible zoonotic reservoir is also reported, which has revived worldwide interest in this disease [5]. To understand the pathogenesis of hepatitis E and to identify new candidate biomarkers that could be helpful in its clinical management, we have probed patients for changes in their plasma peptidome by mass spectrometry. The application of mass spectrometry to proteomics [6] and peptidomics [7] holds considerable promise for the discovery of new bioactive molecules and for elucidating biochemical regulatory networks.

Endogenous peptides have already been established as messengers, hormones or cytokines in many physiological processes. In addition, peptides can be derived from the turnover of blood or tissue proteins. Alterations in peptide levels under disease conditions implicate this class of molecules as potential biomarkers. A limited number of studies have concentrated on naive, circulating peptides, also referred to as peptidome (i.e. the low molecular weight proteome) in blood [8-10]. We have applied a magnetic bead based separation strategy to enrich hydrophobic peptides from plasma, which are then analyzed by MALDI-TOF. Endogenous plasma peptides from patients with acute hepatitis E were compared to healthy controls and differential pattern analysis was performed. The differences have identified discriminating marker peptides for acute HEV infection and these are discussed with a view to better understand disease pathogenesis.

## Methods

### Materials

Acetonitrile (ACN; HPLC grade) was obtained from SD Fine Chemicals (New Delhi, India). Trifluoroacetic acid (TFA) was purchased from Sigma-Aldrich (St. Louis, USA). The peptide and protein calibrants, and α-cyano-4-hydroxycinnamic acid (MALDI-MS matrix) were purchased from Bruker Daltonics, Germany. The matrix was prepared as 10 mg/ml α-cyanohydroxycinnamic acid in ACN/0.1% TFA (1:2). For magnetic bead preparations, we used RPC-18 Dynal Beads and Dynal MPC magnetic stand from Invitrogen (New York, USA). Two calibration standards were used for external calibration of the spectra generated with the reflectron positive (RP) mode containing the following peptide mass (monoisotopic): (M+H)^+ ^Bradykinin (1-7) - 755.39916; angiotensin II - 1046.54180; angiotensin I - 1296.684780; substance P - 1347.735430; bombesin - 1619.822350; renin substrate - 1758.932610; ACTH_clip (1-17) - 2093.086170; ACTH_clip (18-39) - 2465.198340; Somatostatin - 3147.47100 and the linear positive (LP) mode containing Insulin (M+H)^+ ^(avg) - 5734.5200, Cytochrome C (M+2H)^2+ ^- 6181.0500; Myoglobin (M+2H)^2+ ^- 8476.6600; Ubiquitin-I (M+H)^+ ^(avg) - 8565.7600; Cytochrome C (M+H)^+ ^(avg) - 12360.9700; Myoglobin (M+H)^+ ^(avg) - 16952.3100.

### Subjects

Specimens were obtained from patients with jaundice at the Gastroenterology Outpatient Clinic or inpatients at the Army Base Hospital, New Delhi, and Sanjay Gandhi Postgraduate Institute of Medical Sciences, Lucknow, India. All the samples were collected from male patients and the mean age was 31 yrs ± 9. All the patients had clinically diagnosed hepatitis with mean bilirubin levels as 10.11 mg/dl, SGOT levels as 504.8 IU/ml, and SGPT levels as 814.09 IU/ml (Table 1). Specimens were also obtained from healthy volunteers. The study was approved by the Human Subjects Ethics Committee at ICGEB and the respective hospitals, and informed consent was obtained prior to sampling. Blood (5-6 ml) was collected in EDTA-coated vacutainers (Becton Dickinson, Maryland, USA), and centrifuged at 800xg for 5 min at 4°C. The plasma was collected and stored in aliquots at -70°C. It was tested for various viral hepatitis markers by enzyme immunoassays (EIA) as follows: HBsAg (Hepalisa, J Mitra and Co. Pvt. Ltd., New Delhi, India), anti-HCV (Hep-Chex-C, XCyton Diagnostic Pvt. Ltd., Bangalore, India), and IgM anti-HEV (HEV IgM ELISA, MP Biomedicals Asia Pacific Pvt. Ltd., Singapore) as per the manufacturer's protocols. Samples positive for IgM anti-HEV and negative for HCV and HBV markers were selected and grouped together as HEV+ samples.

**Table 1:** Epidemiological data of the patients (P) and healthy controls (N) recruited in the study.

Patient ID	Age (years)	Sex	HEV IgM	HBsAg	HCV IgG	Bilirubin (mg/dl)	SGOT (IU/ml)	SGPT (IU/ml)
P1	58	Male	**_+_**	**-**	**-**	19.1	41	71

P2	39	Male	**_+_**	**-**	**-**	ND	ND	ND

P3	21	Male	**_+_**	**-**	**-**	2.9	ND	ND

P4	14	Male	**_+_**	**-**	**-**	9.4	620	934

P5	25	Male	**_+_**	**-**	**-**	9.5	280	476

P6	24	Male	**_+_**	**-**	**-**	4.6	86	172

P7	22	Male	**_+_**	**-**	**-**	9.8	106	192

P8	26	Male	**_+_**	**-**	**-**	14	386	820

P9	30	Male	**_+_**	**-**	**-**	8	292	640

P10	22	Male	**_+_**	**-**	**-**	5.8	230	510

P11	30	Male	**_+_**	**-**	**-**	9.6	193	334

P12	35	Male	**_+_**	**-**	**-**	4.6	116	328

P13	33	Male	**_+_**	**-**	**-**	6.4	195	393

P14	24	Male	**_+_**	**-**	**-**	8.5	810	1040

P15	39	Male	**_+_**	**-**	**-**	4.1	202	265

P16	31	Male	**_+_**	**-**	**-**	9.1	356	470

P17	24	Male	**_+_**	**-**	**-**	8.2	219	348

P18	36	Male	**_+_**	**-**	**-**	17	1453	1503

P19	34	Male	**_+_**	**-**	**-**	20.9	1008	1170

P20	29	Male	**_+_**	**-**	**-**	12.8	1956	2696

P21	23	Male	**_+_**	**-**	**-**	12.5	69	176

P22	35	Male	**_+_**	**-**	**-**	5.2	464	1476

P23	40	Male	**_+_**	**-**	**-**	10.4	159	340

P24	36	Male	**_+_**	**-**	**-**	6.1	2309	4266

P25	51	Male	**_+_**	**-**	**-**	24.3	61	104

N1	45	Male	**-**	**-**	**-**	ND	ND	ND

N2	30	Male	**-**	**-**	**-**	ND	ND	ND

N3	33	Male	**-**	**-**	**-**	ND	ND	ND

N4	31	Male	**-**	**-**	**-**	ND	ND	ND

N5	27	Male	**-**	**-**	**-**	ND	ND	ND

N6	31	Male	**-**	**-**	**-**	ND	ND	ND

N7	27	Male	**-**	**-**	**-**	ND	ND	ND

N8	28	Male	**-**	**-**	**-**	ND	ND	ND

N9	31	Male	**-**	**-**	**-**	ND	ND	ND

N10	28	Male	**-**	**-**	**-**	ND	ND	ND

ND: Not done

### Peptidome Separation

Dynal beads are supermagnetic, monosized polymer particles with attached bioreactive molecules, which lead to specific surface chemistries that are useful for the separation of biomolecules. The chemical specificities of these beads include reverse phase (RPC18), strong or weak cation exchange (SCX or WCX), and strong anion exchange (SAX). The ligand on RPC18 binds to non-polar amino acids of proteins or peptides through strong hydrophobic adsorption interactions and are eluted using an organic solvent e.g. acetonitrile.

Dynal RPC 18 beads were resuspended thoroughly to obtain a homogeneous suspension. Of these, 20 μL (0.25 mg) were transferred to a tube and placed on a magnet (Dynal MPC) for 1-2 min. The supernatant was removed by aspiration while the tube remained on the magnet. The tube was removed, 100 μL of washing solution (0.1% Trifluoroacetic acid) was applied along the inside of the tube where the beads were collected and resuspended. This step was repeated for a total of 3-4 washes, after which 10 μL of washing solution was added and the beads were resuspended. Five μL of plasma sample was added to the vial containing pre-washed beads, 15 μL more of the washing solution was added and the contents were mixed using a pipette. It was left at room temperature for 2 min to allow the peptides to adsorb to the beads and the tube was placed on the magnet. When the beads were at the tube wall and the liquid was clear, the supernatant was removed and discarded. The tube was removed from the magnet and 50 μL of washing solution was added and mixed. The beads were washed with three changes of washing solution and the supernatant discarded after magnetic separation. The beads were finally resuspended in 6 μL desorption solution (50% acetonitrile), incubated for 2 min at room temperature and the eluate was removed after magnetic separation from the beads.

### MALDI -TOF/TOF Analysis

For this analysis, 1 μL of the eluate was mixed with 1 μL of MALDI-MS matrix and spotted onto a MALDI ground steel target plate (Bruker Daltonics, GmbH). Each sample was spotted in triplicate on the plate and was left to dry for 30 min. Samples and calibration standards with the same matrix composition were spotted adjacent to each other on the target plate for optimal calibration. Spectra generated with the reflectron positive (RP) mode and the linear positive (LP) mode were externally calibrated with the calibration standards as explained in the 'Materials' section. Measurements were performed using an Ultraflex MALDI-TOF/TOF instrument (Bruker Daltonics, GmbH), in the positive ion linear and reflector mode with the accelerating voltage of 25 kV (Ion source 1) and 21.85 kV (Ion source 2) respectively. The laser wavelength and frequency was 337 nm and 67-100 Hz and the percentage was set to 25%. The detector range was set at 400-4000 Da for the reflectron positive mode and 1000-10000 Da for the linear positive mode. Final mass spectra were produced by averaging 1500 laser shots taken at five different positions within each spot. It was processed using the AutoXecute tool of the Flex Control acquisition software. For data analysis, Bruker Daltonics ClinPro Tools (CPT) Software (v2.0) was applied. The CPT workflow starts by spectra loading of two selected classes (HEV+ cases and healthy controls). The CPT software package included an automated raw data pre-treatment workflow, comprising baseline subtraction with 80% baseline flatness (Convex Hull v3), normalization of spectra according to the total ion count and an alignment of peaks with threshold of signal-to-noise ratio (S/N) >5 for peaks and Savitzky-Golay smoothing (1 cycle, m/z range = 5). Peaks with Kruskal-Wallis p-value > 0.1 were discarded. Peak statistics were done using Genetic Algorithm. Finally, the software provided a list of peaks sorted along the statistical difference between two classes, which was used for further data analysis.

### MS/MS based sequencing and identification

For MS/MS based sequencing and identification of proteins from the peptides, the precursor peptide ions were fragmented in positive mode using the MALDI-MS/MS sequencing method. The accelerating voltages were 8.00 kV and 7.15 kV for ion sources 1 and 2, respectively. The reflector 1 and 2 were set to 29.5 kV and 13.9 kV respectively, with Lift 1 and 2 respectively set at 19 kV and 2.8 kV. Calibration was done using angiotensin II and substance P as precursor mass. Fragmentation was done using laser induced dissociation (LID), the fragmented peptide was analysed using Flex Analysis software v3.0 and database search was done using Biotools software v3.2 http://www.bdal.com/products/software/biotools/overview.html. The database search parameters were set as described, the taxonomy was set to *Homo sapiens *and fragment masses were searched in the National Centre for Biotechnology Information database with MS tolerance of 0.1 Da and MS/MS tolerance of 0.5 Da.

## Results

**Figure 1:**
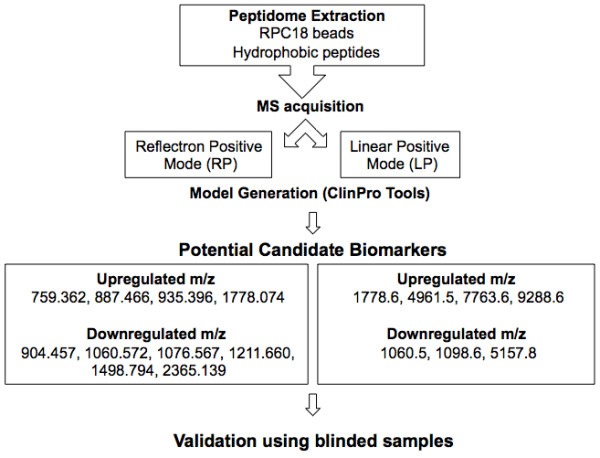
**Schematic representation of peptidome enrichment and analysis**. The methodology used for the enrichment and analysis of plasma peptides is shown. After enrichment of hydrophobic peptides using RPC-18 magnetic beads, the peptide fraction was subjected to mass spectrometry using two different acquisition modes. ClinPro Tools was used for model generation using pattern analysis of the peptide mass fingerprints of patients or controls and the differentiating peaks were identified using MS/MS. Following the discovery phase, different blinded samples were used for validation.

A total of 40 subjects, including 30 patients with acute hepatitis E and 10 healthy controls were recruited for the study. The plasma samples (5 μL of each sample), were processed with Dynalbeads-RPC18 for enrichment of the hydrophobic peptides as described in Methods. Peptide mass fingerprints (PMF) of each sample were generated in the mass range of 400-10000 Da in the two different acquisition modes. The PMFs were loaded into ClinPro Tools (CPT) and processed (Figure 1).

### Model generation and validation

**Figure 2:**
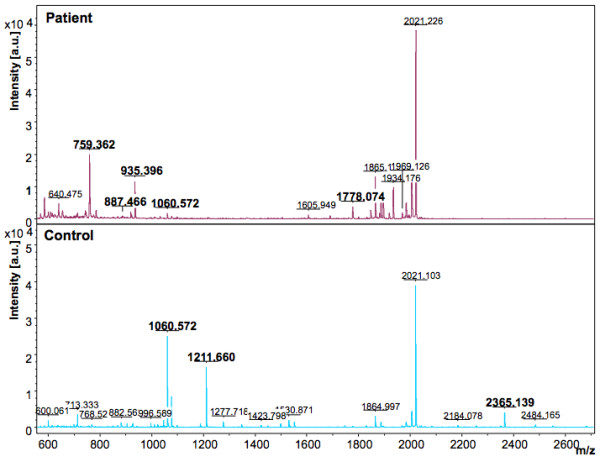
**Representative mass spectra showing peaks in the Reflectron Positive (RP) mode**. The upper and lower panels show peak patterns for representative plasma samples from HEV patients and healthy controls, respectively. Peaks that are upregulated or down regulated in patients and controls in the m/z range of 600 to 2600 are shown. The peaks are marked at their respective m/z values; numbers in bold indicate the discriminating peaks. Averaged mass spectra were generated by ClinPro Tools software (V2.0) using spectra showing the highest number of peaks and signal-to-noise ratios. All spectra were baseline-subtracted, smoothed, normalised and realigned.

**Figure 3:**
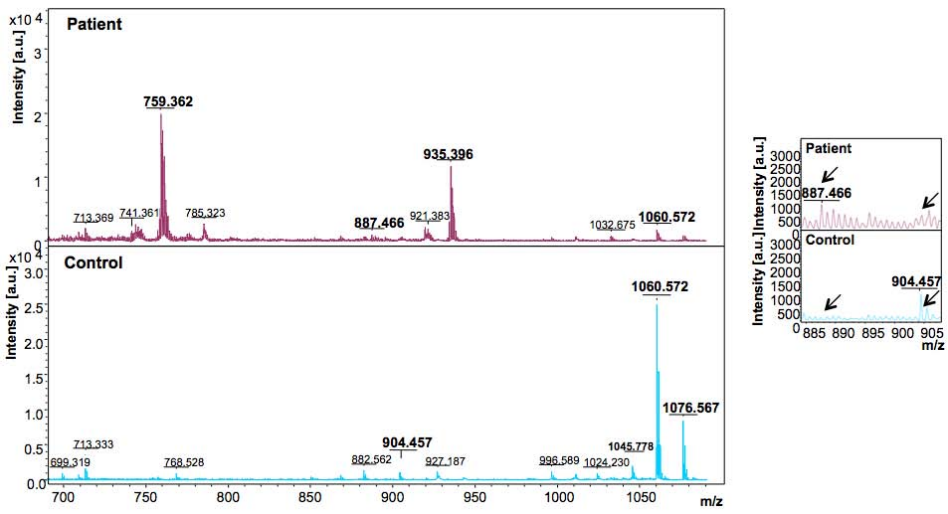
**Representative mass spectra showing peaks in the Reflectron Positive (RP) mode**. The upper and lower panels show peak patterns for representative plasma samples from HEV patients and healthy controls, respectively. Peaks in the m/z range of 700 to 1100 are shown. The upper and lower panels show peak patterns for representative plasma samples from HEV patients and healthy controls, respectively. Averaged mass spectra were generated by ClinPro Tools software (V2.0) using spectra showing the highest number of peaks and signal-to-noise ratios. All spectra were baseline-subtracted, smoothed, normalised and realigned. Insets show discriminating peaks not readily apparent at the scale of the main figure.

**Figure 4:**
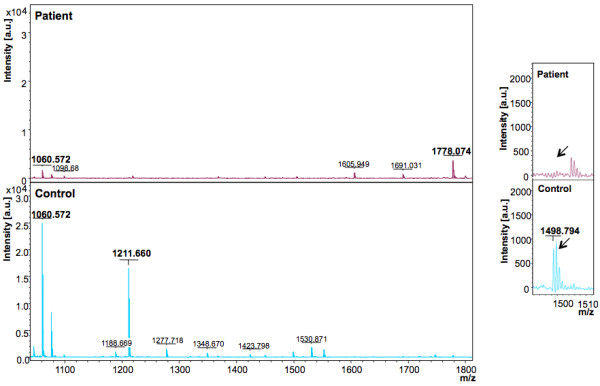
**Representative mass spectra showing peaks in the Reflectron Positive (RP) mode**. The upper and lower panels show peak patterns for representative plasma samples from HEV patients and healthy controls, respectively. Peaks in the m/z range of 1050 to 1800 are shown. The upper and lower panels show peak patterns for representative plasma samples from HEV patients and healthy controls, respectively. Averaged mass spectra were generated by ClinPro Tools software (V2.0) using spectra showing the highest number of peaks and signal-to-noise ratios. All spectra were baseline-subtracted, smoothed, normalised and realigned. Insets show discriminating peaks not readily apparent at the scale of the main figure.

**Figure 5:**
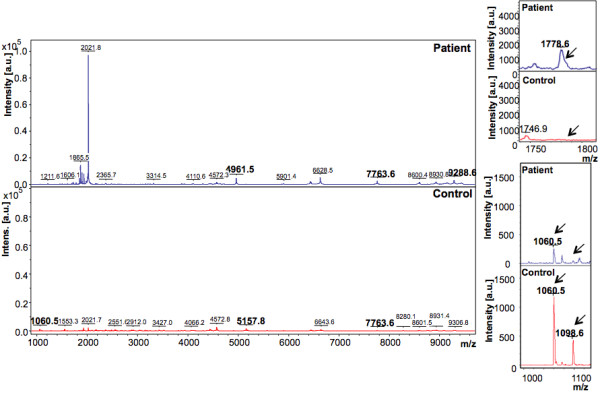
**Representative mass spectra showing peaks in the Linear Positive (LP) mode**. The upper and lower panels show peak patterns for representative plasma samples from HEV patients and healthy controls, respectively. Peaks that are upregulated or down regulated in patients and controls in the m/z range of 1000 to 10000 are shown. The peaks are marked at their respective m/z values; numbers in bold indicate the discriminating peaks. Averaged mass spectra were generated by ClinPro Tools software (V2.0) using spectra showing the highest number of peaks and signal-to-noise ratios. All spectra were baseline-subtracted, smoothed, normalised and realigned. Insets show discriminating peaks not readily apparent at the scale of the main figure.

**Figure 6:**
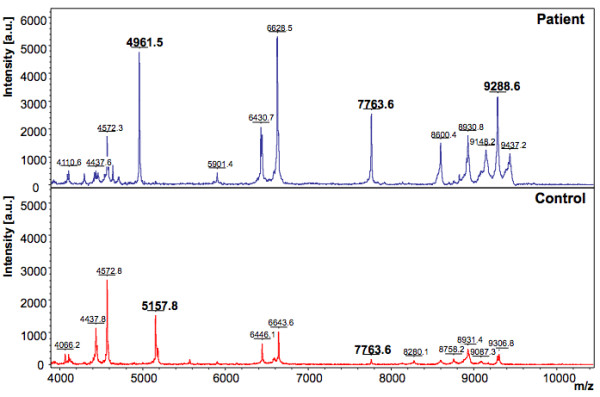
**Representative mass spectra showing peaks in the Linear Positive (LP) mode**. The upper and lower panels show peak patterns for representative plasma samples from HEV patients and healthy controls, respectively. Peaks that are upregulated or down regulated in patients and controls in the m/z range of 4000 to 10000 are shown. The peaks are marked at their respective m/z values; numbers in bold indicate the discriminating peaks. Averaged mass spectra were generated by ClinPro Tools software (V2.0) using spectra showing the highest number of peaks and signal-to-noise ratios. All spectra were baseline-subtracted, smoothed, normalised and realigned.

**Figure 7:**
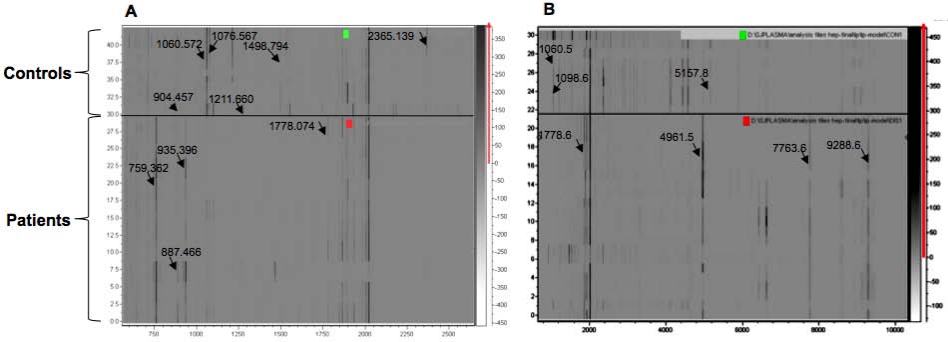
**Density Plot of the peptide profiles**. Density plots (pseudo gel view) of the plasma peptide profiles of HEV patients and healthy controls are shown. Differentiating peaks that are identified using the (A) RP mode, and (B) LP mode are marked with arrows. Upper panels are the gel views of control samples and lower panels represent the patient samples. The MS profiles were generated by ClinPro Tools software (V2.0) and were baseline-subtracted, smoothed, and normalised.

In the discovery phase, two models were generated based on the acquisition modes, for the peptidome profiling of HEV patient plasma cross-tested against healthy controls. The Reflectron Positive (RP) Model was generated for peaks in the mass range of 400-4000 Da using 10 HEV+ samples and 4 healthy controls, designated as Class 1 and 2, respectively for the Clinpro analysis. The model which could correctly classify disease and control samples was generated using the following parameters: maximum number of best peaks - 20; number of generations - 50; number of nearest neighbours - 3; mutation rate - 0.20. Analysis was performed and CPT generated a table ranking the mass spectral peaks in order of their *p*-values calculated using the statistical tests: P-value of student 't' test and analysis of variance (p value tta) and P-value of Wilcoxon/Kruskal-Wallis test (p value wkw). According to these tests and manual validation, 31 peaks were identified as statistically significant between the two classes. Four peaks with m/z of 759.362, 887.466, 935.396 and 1778.074 that were upregulated in hepatitis E patient plasma and six peaks with m/z of 904.457, 1060.572, 1076.567, 1211.660, 1498.794 and 2365.139 that were downregulated in HEV infection were selected (Figure 2, 3, 4). These 10 peaks were then used for further validation. In the validation phase, 6 specimens each from the two classes were tested in a blinded manner; these samples were different from those used for model generation. Data analysis using Clinpro software and manual inspection could efficiently distinguish between the two classes of patients and controls with a recognition capability of 96%. The other mode of data acquisition was the linear positive mode. The Linear Positive (LP) Model for peaks in the range of 1000-10000 Da was generated using 11 HEV+ and 4 healthy control samples, again in a blinded manner with new samples. Analysis was done with genetic algorithm to identify the discriminating peaks. Using the statistical tests and manual validations, a total of 102 discriminating peaks were identified. Of these, 7 peaks were selected for the validation phase. These included 4 peaks that were upregulated in hepatitis E patient plasma with m/z values of 1778.6, 4961.5, 7763.6 and 9288.6, and 3 peaks that were downregulated in HEV infection with m/z values of 1060.5, 1098.6 and 5157.8 (Figure 5, 6). For the validation phase, 6 samples each in the two classes were studied in a blinded manner. Manual inspection of peaks and Clinpro analysis could efficiently distinguish between patients and healthy individuals with a recognition capability of 100%. In the RP mode, hepatitis E patients and healthy controls showed discriminating peaks in the m/z ranges of 600 to 2600 Da (Figure 2), 700 to 1100 Da (Figure 3) and 1050 to 1800 Da (Figure 4). The discriminating peak profiles acquired by the LP mode are shown in the m/z ranges of 1000 to 10000 Da (Figure 5) and 4000 to 10000 Da (Figure 6). The smaller discriminating peaks are shown in insets in Figure 3, 4 and 5. Density plots showing differentially regulated peptides identified by the RP and LP modes are also shown in Figure 7A and 7B, respectively.

### Receiver Operating Characteristic Curve

**Figure 8:**
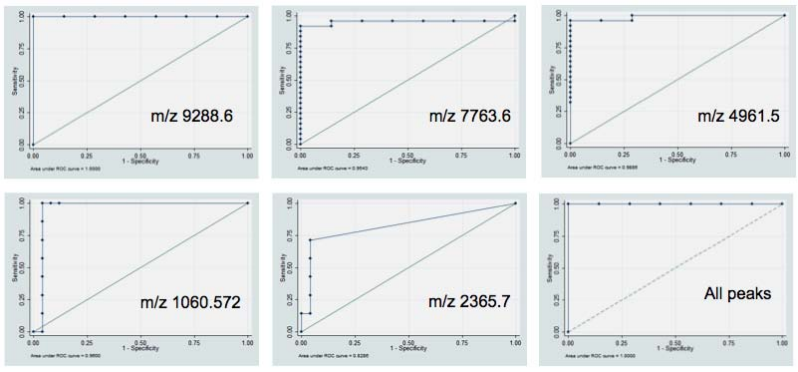
**Receiver Operator Characteristics Curve (ROC) for the discriminating peaks**. The Area under the curve (AUROC) for peaks with m/z values indicated on the graphs is shown. These are 1.00 for m/z 9288.6 and 0.954 for m/z 7763.6 and 0.989 for m/z 4961.5. Peak m/z 1060.572 was identified as Bradykinin with an AUROC value of 0.960; peak m/z 2365.139 was identified as Kininogen with an AUROC value of 0.829. The AUROC value for all 5 peaks taken together is 1.0.

The receiver operating characteristic (ROC) curve plots correct identifications (i.e. sensitivity) and misidentifications (1-specificity) when using a diagnostic test, and provides a graphical representation of the latter's effectiveness in discriminating between two groups. The area under the ROC curve (AUROC) is a reflection of test reliability in discriminating two sets, these being HEV patients and healthy controls in our case. The ROC curves were generated using intensity data from 25 patients and 7 controls. The intensity value of each peak was taken into account. Each sample was subjected to MS acquisition in triplicates and the intensity of the same peak in at least two of the spectra was averaged and used for the ROC calculation. The AUROC values above 0.75 are considered to provide good discrimination [11]. Based on the quantitative data of peak intensities, the AUROC for peaks with m/z values of 9288.6, 7763.6 and 4961.5, which increase in HEV patient plasma were found to be 1.00, 0.954 and 0.989 respectively with 95% confidence intervals of 1.00-1.00 for peak m/z 9288.6, 0.876-1.033 for peak m/z 7763.6 and 0.960-1.017 for peak m/z 4961.5 (Figure 8) (Intercooled Stata 9.2 for Windows; Stata Corp, College Station, TX). Two peaks with m/z values of 1060.572 and m/z 2365.139, which decrease in the plasma of hepatitis E patients, had AUROC values of 0.960 and 0.829 respectively with 95% confidence intervals of 0.883-1.037 and 0.619-1.038, respectively (Figure 8). The ROC curves were also generated for all possible combinations of two markers each (n = 10), three markers each (n = 10), four markers each (n = 5), and for all the 5 markers. All the 26 possible combinations had very high AUROC values, with the minimum being 0.963 (95% CI = 0.890-1.000); 17 of these combinations, including the one with all 5 markers (Figure 8) had area under curve of 1.0. Thus, these five peaks discriminate HEV patients from healthy individuals with high sensitivity and specificity.

It may be noted that the number of data points are not the same on each ROC curve (Figure 8). This is not equal to the number of specimens studied, but is equal to the number of pairs of (1-specificity) and sensitivity possible with different cut-offs. The highest number possible for such data points is the number of specimens studied. However, if some specimens have identical results, then the number of data points for ROC curve is fewer. Since one of the peaks (m/z 9288.6) shows perfect separation between patients and controls, there may not be much point in adding other peaks to it. This might result in an over-fitted model. However, an alternative view may be that the observed ROC curves are based on the particular sample studied. If one were to study other groups of patients and controls, the ROC curves for those could deviate from the observed curves (e.g. area-under-curve for each ROC curve have a standard deviation around the observed value). The combined ROC curve, being based on five peaks, is likely to have a lower variance.

### Identification of peptides using MS-MS

Potential biomarker peptides discovered by the Clinpro Tools software were subjected to in-depth fragment analysis using the TOF/TOF capabilities of the Ultraflex. BioTools software was also used for peptide identification by database search. Some of the peptides that were downregulated in acute HEV infection were identified as: m/z 904.457, Bradykinin; m/z 1060.572, Bradykinin; m/z 1076.567, Bradykinin; m/z 1098.6, Complement C3; m/z 1211.660, Complement C3f; m/z 1498.794, Complement C4; m/z 2365.139, Kininogen. Peptides with m/z 759.362, 887.466, 935.396, 4961.5, 7763.6 and 9288.6, which were predominantly present in the plasma of HEV infected patients, could not be identified by MS/MS. This could be because the peptide mass observed might belong to a degraded or modified part of a novel or uncharacterised protein. Searching the MALDI-TOF/TOF spectrum against the mammalian and the viral database and with no restriction in taxonomy through NCBI also did not result in a reliable identification of these peptides.

## Discussion

While HEV mainly causes self-limiting sporadic and epidemic acute viral hepatitis, a more severe fulminant hepatic failure (FHF) is seen in some patients [12]. Further, chronic liver disease patients superinfected with HEV also show higher morbidity and mortality [13]. During pregnancy, hepatitis E is associated with increased frequency of severe liver disease, mortality and adverse fetal outcomes than disease caused by other hepatitis viruses [14]. An increase in estrogen, progesterone and β-HCG levels was observed in HEV-positive pregnant patients with FHF compared to HEV-negative patients and controls [15]. This alteration in hormones may enhance viral replication, leading to severe liver disease during pregnancy [16]. Severe liver damage following HEV infection has also been correlated to the absence of NF-κB p65 subunit [17]. These observations suggest that the severity of HEV infection is influenced by the host response; identifying disease related host signatures would thus be important to understand disease pathogenesis and improving prognosis.

The plasma peptidome contains protein fragments derived from cells and tissues. While some researchers have dismissed the peptidome as 'biological trash', recent work has indicated that it may shed light on pathophysiological perturbations to host physiology and biochemical regulatory networks, and may lead to the identification of diagnostic biomarkers [8-10,18].

Since blood plasma is a complex body fluid [19], we used a magnetic bead based fractionation strategy to simplify the plasma peptidome. Beads coated with C18 were used to enrich hydrophobic peptides from each sample, for which a peptide mass fingerprint (PMF) was generated. The analysis led to an averaged peptide pattern for controls and tests (HEV samples) and a specific pattern representative of hepatitis E was discovered. Analysis using different acquisition modes identified 14 unique peaks that were differentially regulated during acute hepatitis E. This peptide pattern was further validated using blinded assessment of other plasma samples and classifying peptides with m/z values of 9288.6, 7763.6, 4961.5, 1060.572 and 2365.139 were identified. Of these, the first three unidentified peptides were increased, while the other two, identified as kininogen and bradykinin, were decreased in the plasma of hepatitis E patients.

The innate immune system is the first line of defence, comprising of cells and mechanisms that defend the host from infection in a non-specific manner. Inflammation is one of the first responses to infection or irritation. Various chemical factors released by injured cells stimulate an inflammatory response and serve to establish a physical barrier against the spread of infection, and promote healing of any damaged tissue following the clearance of pathogens [20]. A number of chemical factors such as histamine, bradykinin, serotonin and prostaglandins are produced during inflammation, which sensitize pain receptors, cause vasodilation at the scene, and attract phagocytes. The kinin forming pathway is activated on endothelial cells and neutrophils when high molecular weight kininogen (HK) is cleaved by plasma kallikrein liberating bradykinin, a potent mediator of inflammation [21]. Another arm of the innate immune system comprises of complement proteins that also link to the adaptive immune response. This cascade is composed of many plasma proteins synthesized by hepatocytes. Thus any perturbation such as a viral infection in the liver should trigger a strong innate response as the first line of host defence. In this study, the levels of complement proteins C3, C3f, C4, and bradykinin and kininogen were found to be lower in the plasma of hepatitis E patients compared to healthy controls. The exact mechanism for this reduction is not understood at this time.

**Figure 9:**
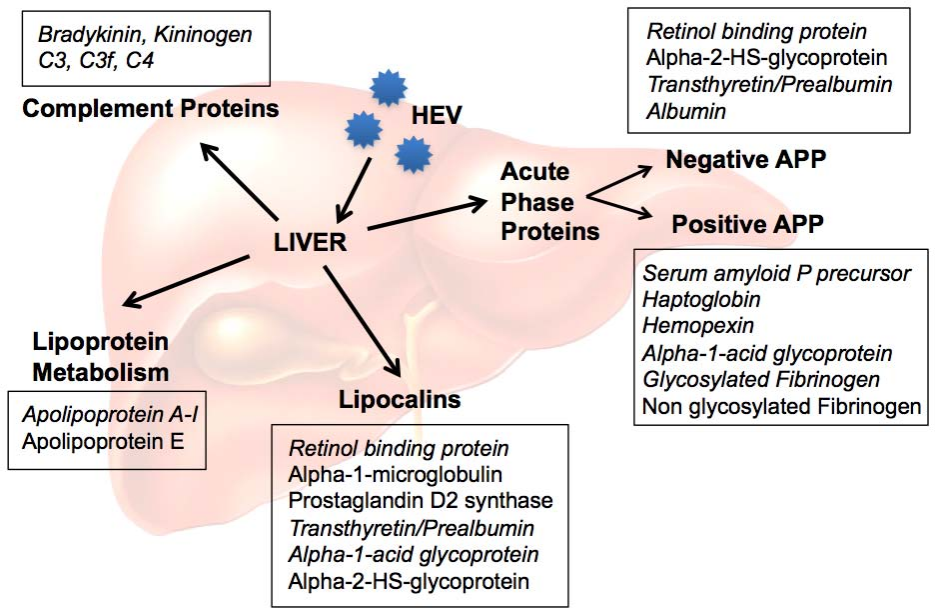
**Differentially regulated proteins in HEV infected patients**. An overview of the various pathways perturbed due to hepatitis E virus infection is presented. The plain and italicized texts indicate increased or decreased expression of the proteins, respectively, in patients compared to controls.

We previously reported profiling the plasma and urine proteomes of hepatitis E patients and identified various proteins that could reliably identify the acute phase of disease [22]. On combining results from that study and the present one, several differentially regulated proteins were identified during acute HEV infection. These belong to different classes namely, acute phase proteins (APP), lipocalins, proteins involved in lipoprotein metabolism and complement proteins (Figure 9). HEV infection modulates the acute phase response, a major inflammatory pathway in the liver as observed from the decrease in positive APPs like haptoglobin, hemopexin, serum amyloid P precursor and alpha-1 acid glycoprotein, and increase in the anti-inflammatory negative APP, alpha-2-HS glycoprotein (or fetuin). A direct effect of the HEV open reading frame 3 (ORF3) protein has been proposed on the expression of APPs [23].

Infection by HEV also results in modulation of the lipocalin family of proteins. These are a group of small extracellular ligand binding proteins with diverse functional and structural properties. These involve retinol transport (retinol binding protein), prostaglandin synthesis (prostaglandin D2 synthase), regulation of cell homeostasis and immune response (alpha-1-microglobulin, alpha-1-acid glycoprotein, transthyretin) [24-26]. In our proteome analysis of urine and plasma from hepatitis E patients, these proteins were differentially modulated [22], as indicated in Figure 9.

Apolipoproteins are lipid binding proteins that transport dietary lipids through the bloodstream from the intestine to liver or endogenously synthesized lipids from the liver to other tissues for storage (adipocytes), metabolism (muscle, heart, lung) or secretion, and play an important role in lipid metabolism. During the acute phase response, there is a decrease in apoA-I, apoA-II and apoC levels [27-29], whereas apoE is increased in some studies [27,30], but decreased in others [31]. We also observed a decrease in apoA-I levels and an increase in apoE levels in the plasma of acute hepatitis E patients compared to healthy controls [22].

The mechanistic details of these changes or a direct involvement of HEV proteins are presently not understood. This study in combination with the earlier analyses of the plasma and urine proteomes further strengthens the observation that HEV suppresses innate responses to establish infection and replicate in the host. This effect is likely to be short-lived since HEV causes only an acute self-limiting infection, except in immunologically compromised hosts where it can also cause a chronic infection [32-34]. This constitutes the first such study on HEV and hepatitis E, which allows insights into viral pathogenesis and also provides biomarkers which can be used to reliably identify hepatitis E patients.

There are some limitations of the present study. We have used only male patients in this study due to the major location of our sampling, and have compared only hepatitis E patients to healthy controls. We cannot therefore say whether the peptide signatures discovered here are specific for hepatitis E or they would also be found in other forms of hepatitis. Further, our separation method utilizes beads that capture only hydrophobic peptides. There could be other potential marker peptides with different chemical properties. Finally, we have validated the discovered signatures using the same separation and mass spectrometric approach instead of another platform. Notwithstanding these limitations, this study provides an important methodological platform for further discovery, which will be used in future to compare fulminant and self-limited hepatitis E patients.

## Conclusions

This study compared the plasma peptidome of hepatitis E patients to healthy controls. Using mass spectrometric analysis of the peptides we have identified a signature comprising of five potential biomarker peaks that could reliably identify patients and controls. We have also identified a number of peptides that are differentially regulated in hepatitis E disease and which are degraded or processed products of some of the key players of host innate immunity. Besides identifying potential biomarker peptides, this study and an earlier one reported from our group [22] have helped increase our understanding of hepatitis E disease pathogenesis and the possible host pathways perturbed by the hepatitis E virus for its own survival.

## Competing interests

The authors declare that they have no competing interests.

## Authors' contributions

ST and SJ planned the experiments; ST and IA carried out the experiments; SS, SK, RA^2^, VSR, KN did the mass spectrometric analyses; VKG and RA provided the clinical samples; RA^5 ^helped with the statistical analyses; ST and SJ wrote the manuscript. All authors read and approved the final manuscript.
